# Supplementary material for: Polyphenol profile of buckwheat honey, nectar and pollen
Source: R Soc Open Sci. 2020 Dec 9;7(12):201576. doi: 10.1098/rsos.201576 (PMC7813236; doi:10.1098/rsos.201576)
Supplement: Table S1 and S2 [file rsos201576supp1.docx]

**Supplementary material**

Polyphenolic profile of buckwheat honey, nectar and pollen

Milica Nešović^1^, Uroš Gašić^2^, Tomislav Tosti^3^, Nikola Horvacki^4^, Branko Šikoparija^5^, Nebojša Nedić^6^, Stevan Blagojević^1^, Ljubiša Ignjatović^7^ and Živoslav Tešić^3^*

*^1^Institute of General and Physical Chemistry, Studentski trg 12-16, Belgrade 11158, Serbia*

*^2^Department of Plant Physiology, Institute for Biological Research “Siniša Stanković”, National Institute of Republic of Serbia, University of Belgrade, Bulevar despota Stefana 142, Belgrade 11060, Serbia;*

*^3^University of Belgrade – Faculty of Chemistry, Studentski trg 12-16, P.O. Box 51, Belgrade 11158, Serbia;*

*^4^Innovation Center, University of Belgrade – Faculty of Chemistry, P.O. Box 51, Belgrade 11158, Serbia;*

*^5^BioSense Institute - Research Institute for Information Technologies in Biosystems, University of Novi Sad, Novi Sad 21101, Serbia;*

*^6^Faculty of Agriculture, Institute for zootehnics, University of Belgrade, Nemanjina 6, Belgrade – Zemun 11080, Serbia;*

^7^University of Belgrade –Faculty of Physical Chemistry, Studentski trg 12-16, Belgrade 11158, Serbia;

*Corresponding author:

Živoslav Lj. Tešić

Address: Studentski trg 12-16, 11158 Belgrade, Serbia

Tel: +381113336733

E-mail: [ztesic@chem.bg.ac.rs](mailto:ztesic@chem.bg.ac.rs)

**Figure caption**

Figure S1. Micrographs of pollen types contributing pollen spectrum with >15%.

**Table captions**

Table S1. Melissopalynological analysis of presence nectraiferous and nectarless species in Serbian (H1-H4) and Polish honey samples (H5, H6).

Table S2. Content of 31 phenolic compounds (mg/kg), TPC (mg GAE/kg) and RSA (%) in buckwheat honey samples from Serbia (H1-H4) and Poland (H5, H6).

**Table S1**. Melissopalynological analysis of presence nectraiferous and nectarless species in Serbian (H1-H4) and Polish honey samples (H5, H6).

| **Type of nectraiferous/nectarless plant** | |  | **Serbian honey** | | | |  | **Polish honey** | |
| --- | --- | --- | --- | --- | --- | --- | --- | --- | --- |
| ***Genus*** | **Family** |  | **H1** | **H2** | **H3** | **H4** |  | **H5** | **H6** |
| *Achilea* type | Asteraceae |  | 0.90 | 0 | 1.09 | 0.31 |  | 0.71 | 1.53 |
| *Ailanthus* | Simaroubaceae |  | 0 | 0 | 0.22 | 0 |  | 0 | 0 |
| *Alnus** | Betulaceae |  | 0 | 0.12 | 0.22 | 0.92 |  | 0.12 | 0 |
| *Amorpha* type | Fabaceae |  | 0 | 0.74 | 19.21 | 3.08 |  | 0 | 0 |
| *Apiaceae* | Apiaceae |  | 0 | 0.12 | 0.66 | 0.92 |  | 0 | 0 |
| *Artemisia** | Asteraceae |  | 0 | 0 | 0 | 0.62 |  | 2.60 | 0.87 |
| *Aster*type | Asteraceae |  | 0.90 | 0.12 | 0 | 0 |  | 0.24 | 0 |
| *Astragalus* type | Fabaceae |  | 4.50 | 6.62 | 2.84 | 6.15 |  | 0.47 | 3.06 |
| *Betula* | Betulaceae |  | 0 | 0 | 0.66 | 1.23 |  | 0 | 0 |
| *Brassica napus* | Brassicaceae |  | 0 | 0 | 0 | 0 |  | 11.24 | 0 |
| *Brassicaceae* | Brassicaceae |  | 0.13 | 0.49 | 0.22 | 0.92 |  | 0 | 27.29 |
| *Carduus* | Asteraceae |  | 0 | 0 | 1.09 | 0.31 |  | 0 | 0 |
| *Cariophilaceae* | Cariophilaceae |  | 0 | 0 | 0 | 0 |  | 0 | 0 |
| *Centaurea cyans* | Asteraceae |  | 0 | 0 | 0 | 0 |  | 1.78 | 0.44 |
| *Centaurea jacea* | Asteraceae |  | 0.13 | 0.37 | 0 | 0 |  | 0 | 0 |
| *Chenopodiaceae** | Amaranthaceae |  | 0 | 0 | 0 | 0 |  | 0.36 | 4.37 |
| *Clematis* | Rannunculaceae |  | 0 | 0 | 5 | 0 |  | 0 | 0 |
| *Convolvulus* | Convolvulaceae |  | 0 | 0 | 0 | 0 |  | 0 | 0.22 |
| *Corylus** | Betulaceae |  | 0 | 0 | 0 | 0.62 |  | 0 | 0 |
| *Cupressaceae** | Cupressaceae |  | 0 | 0 | 1.75 | 0.92 |  | 0 | 0 |
| *Echium* | Boraginaceae |  | 6.95 | 0.25 | 0 | 0 |  | 0 | 0 |
| *Epilobium* | Onagraceae |  | 0.26 | 0 | 0.22 | 0 |  | 0 | 0 |
| *Euphorbia* | Euphorbiaceae |  | 0 | 0 | 0.22 | 0 |  | 0 | 0 |
| *Fagopyrum* | Polygonaceae |  | 0.77 | 1.10 | 10.26 | 4.00 |  | 18.70 | 20.52 |
| *Fenestrate* | Asteraceae |  | 0.26 | 0.12 | 1.09 | 3.08 |  | 0.59 | 7.21 |
| *Filipendula* | Rosaceae |  | 1.42 | 0.49 | 18.12 | 29.85 |  | 0.12 | 0.66 |
| *Fraxinus americana/pensylvanica** | Oleaceae |  | 80.44 | 86.64 | 0.22 | 0 |  | 49.70 | 6.77 |
| *Fraxinus ornus** | Oleaceae |  | 0.13 | 0 | 0 | 0 |  | 0 | 0 |
| *Gleditschia* | Fabaceae |  | 0 | 0 | 1.31 | 0 |  | 0 | 0 |
| *Hedera* | Araliaceae |  | 0 | 0 | 0 | 0.62 |  | 0 | 0 |
| *Helianthus* | Asteraceae |  | 0 | 0 | 0.22 | 0.62 |  | 0.59 | 0.22 |
| *Hypericum* | Hypericaceae |  | 0.26 | 0.61 | 0 | 0.31 |  | 0.12 | 0.22 |
| *Juniperus** | Cupressaceae |  | 0.13 | 0.74 | 0 | 0 |  | 0.59 | 0.22 |
| *Lamiaceae* S type | Lamiaceae |  | 0.51 | 0.49 | 0.44 | 0.31 |  | 0 | 0 |
| *Ligustrum* | Oleaceae |  | 0 | 0 | 0 | 0.31 |  | 0 | 0 |
| *Loranthus* type | Loranthaceae |  | 0 | 0 | 0 | 0.92 |  | 0 | 0 |
| *Lotus* | Fabaceae |  | 0.51 | 0.12 | 0 | 0.92 |  | 0 | 0 |
| *Ostrya** | Betulaceae |  | 0.13 | 0 | 0.66 | 0 |  | 0 | 0 |
| *Phacelia* | Boraginaceae |  | 0 | 0 | 0 | 0 |  | 4.62 | 0 |
| *Plantago** | Plantaginaceae |  | 0 | 0.12 | 1.97 | 2.77 |  | 0.24 | 1.09 |
| *Poaceae** | Poaceae |  | 0 | 0 | 0.66 | 0.62 |  | 0 | 0.66 |
| *Poligonum* | Polygonaceae |  | 0 | 0 | 0 | 0 |  | 0 | 0.44 |
| *Populus** | Salicaceae |  | 0 | 0 | 0 | 0.31 |  | 0 | 0 |
| *Rhamnus* type | Rhamnaceae |  | 0 | 0 | 2.40 | 4.92 |  | 0 | 0 |
| *Robinia* | Fabaceae |  | 0 | 0 | 1.75 | 2.77 |  | 0 | 0 |
| Rosaceae (*Prunus* type) | Rosaceae |  | 0.51 | 0 | 2.40 | 7.08 |  | 1.30 | 1.31 |
| *Rubus* | Rosaceae |  | 0.39 | 0.12 | 14.85 | 10.77 |  | 0.71 | 17.69 |
| *Rumex** | Polygonaceae |  | 0 | 0 | 0 | 2.15 |  | 0.47 | 1.53 |
| *Salix* | Salicaceae |  | 0 | 0 | 0.44 | 0.62 |  | 0 | 0 |
| *Sambucus nigra* | Capryfoliaceae |  | 0 | 0 | 0.22 | 0.31 |  | 0 | 0 |
| *Senecio* | Asteraceae |  | 0 | 0 | 0.44 | 0 |  | 2.25 | 1.75 |
| *Teucrium* | Lamiaceae |  | 0 | 0 | 6.77 | 3.69 |  | 0 | 0 |
| *Tilia* | Malvaceae |  | 0 | 0 | 0.66 | 2.77 |  | 0.12 | 0.22 |
| *Trifolium pratense* | Fabaceae |  | 0.39 | 0.37 | 1.97 | 4.00 |  | 1.54 | 1.53 |
| *Ulmus** | Ulmaceae |  | 0 | 0 | 0 | 0.31 |  | 0 | 0 |
| *Urtica** | Urticaceae |  | 0.13 | 0 | 0 | 0 |  | 0 | 0 |
| *Vicia* | Fabaceae |  | 0 | 0.25 | 0 | 0 |  | 0.71 | 0 |
| *Viola* | Violaceae |  | 0.26 | 0 | 0 | 0 |  | 0 | 0.22 |
| *Zea** | Poaceae |  | 0 | 0 | 0 | 0 |  | 0.12 | 0 |
| Sum |  |  | 100 | 100 | 100 | 100 |  | 100 | 100 |

Pollen frequency classes: P-"Predominant pollen" (more than 45% of pollen grains counted), S-"Secondary pollen" (16-45%); I-"Important minor pollen" (3-15%); M-"Minor important pollen" (less than 3%).* Nectarless species.

**Table S2**. Content of 31 phenolic compounds (mg/kg), TPC (mg GAE/kg) and RSA (%) in buckwheat honey samples from Serbia (H1-H4) and Poland (H5, H6).

| Content of 31 phenolic compounds (mg/kg), TPC (mgGA/kg) and RSA (%) in buckwheat honey samples from Serbia (1-4) and Poland (5,6). | | | | | | | | | | |
| --- | --- | --- | --- | --- | --- | --- | --- | --- | --- | --- |
| No | Phenolic compound | **Serbia** | | | | |  | **Poland** | | |
|  |  | **H1** | **H2** | **H3** | **H4** | **Mean ± SD** |  | **H5** | **H6** | **Mean ± SD** |
| 1 | Quercetin | 2.2206 | 3.2332 | 3.8906 | 1.6997 | 2.761 ± 0.986 |  | 26.3997 | 2.9557 | 14.678 ± 16.577 |
| 2 | Kaempferol | 0.5851 | 0.7271 | 0.7111 | 0.2938 | 0.579 ± 0.201 |  | 1.0238 | 0.8205 | 0.922 ± 0.144 |
| 3 | Galangin | 2.6481 | 3.0755 | 2.2044 | 0.8132 | 2.185 ± 0.981 |  | 0.5914 | 0.3726 | 0.482 ± 0.155 |
| 4 | Kaempferide | 0.0796 | 0.0864 | 0.0275 | 0 | 0.048 ± 0.042 |  | 0.1799 | 0.0973 | 0.139 ± 0.058 |
| 5 | Apigenin | 0.6039 | 0.8949 | 0.3455 | 0.1515 | 0.499 ± 0.323 |  | 0.2097 | 0.1879 | 0.199 ± 0.015 |
| 6 | Chrysin | 3.7769 | 4.2830 | 3.4321 | 1.6963 | 3.297 ± 1.123 |  | 0.7900 | 0.7204 | 0.755 ± 0.049 |
| 7 | Acacetin | 0 | 0 | 0 | 0 | 0 |  | 0.1188 | 0.0814 | 0.100 ± 0.026 |
| 8 | Luteolin | 0.3694 | 0.4219 | 0.1082 | 0.0500 | 0.237 ± 0.186 |  | 0.0378 | 0.0723 | 0.055 ± 0.024 |
| 9 | Genkwanin | 0 | 0 | 0 | 0 | 0 |  | 0.1385 | 0.0911 | 0.115 ± 0.034 |
| 10 | Pinocembrin | 6.0022 | 6.4967 | 5.0917 | 2.6685 | 5.065 ± 1.700 |  | 1.1658 | 0.8831 | 1.024 ± 0.200 |
| 11 | Naringenin | 0.1227 | 0.1433 | 0.0829 | 0.0377 | 0.097 ± 0.047 |  | 0.2021 | 0.1938 | 0.198 ± 0.006 |
| 12 | Eriodictyol | 0 | 0.2035 | 0.1026 | 0.0861 | 0.098 ± 0.083 |  | 0 | 0.0864 | 0.043 ± 0.061 |
| 13 | Genistein | 0.2153 | 0.3136 | 0 | 0.0391 | 0.142 ± 0.148 |  | 0 | 0 | 0 |
|  | **Sum of flavonoids** | **16.624** | **19.879** | **15.997** | **7.536** | **15.01 ± 5.27** |  | **30.857** | **6.563** | **18.71 ± 17.18** |
| 14 | *p*-Hydroxybenzoic acid | 3.8421 | 3.9913 | 7.4047 | 2.7905 | 4.507 ± 2.004 |  | 31.5745 | 15.9662 | 23.770 ± 11.037 |
| 15 | Protocatechuic acid | 0.0914 | 0.0816 | 0.5655 | 0.1914 | 0.232 ± 0.227 |  | 0 | 0 | 0 |
| 16 | Vanillic acid | 1.4216 | 1.4767 | 0.9610 | 0.4349 | 1.074 ± 0.484 |  | 0.8681 | 1.0729 | 0.971 ± 0.145 |
| 17 | p-Hydroxyphenylacetic acid | 1.0342 | 1.1279 | 0.9485 | 0.4110 | 0.880 ± 0.321 |  | 2.7621 | 0.9031 | 1.833 ± 1.315 |
| 18 | Caffeic acid | 5.1628 | 4.7716 | 3.0428 | 1.7931 | 3.693 ± 1.566 |  | 1.6230 | 1.4779 | 1.550 ± 0.103 |
| 19 | 5-*O*-Caffeoylquinic acid | 0.2914 | 0.4560 | 0.1133 | 0.0610 | 0.230 ± 0.180 |  | 0.4093 | 0.1276 | 0.268 ± 0.199 |
| 20 | *p*-Coumaric acid | 2.6375 | 2.6893 | 4.0326 | 1.9203 | 2.820 ± 0.881 |  | 15.5523 | 11.1211 | 13.337 ± 3.133 |
| 21 | Ferulic acid | 3.7280 | 3.9435 | 2.5104 | 1.3847 | 2.892 ± 1.186 |  | 2.7642 | 2.2729 | 2.519 ± 0.347 |
| 22 | Sinapic acid | 0 | 0 | 0.0798 | 0.0265 | 0.027 ± 0.038 |  | 0.0634 | 0 | 0.032 ± 0.045 |
|  | **Sum of phenolic acids** | **18.209** | **18.538** | **19.659** | **9.013** | **16.36 ± 4.93** |  | **55.617** | **32.942** | **44.28 ± 16.03** |
| 23 | Apigenin 7-O-glucoside (Apigetrin) | 0 | 0 | 0 | 0 | 0 |  | 0 | 0 | 0 |
| 24 | Apigenin 8-C-glucoside (Vitexin) | 0 | 0 | 0 | 0 | 0 |  | 0.0793 | 0 | 0.040 ± 0.056 |
| 25 | Kaempferol 7-O-glucoside | 0.0437 | 0.0732 | 0.0376 | 0 | 0.039 ± 0.030 |  | 0.0384 | 0.0236 | 0.031 ± 0.010 |
| 26 | Isorhamnetin 3-O-glucoside | 0 | 0.0313 | 0.0248 | 0 | 0.014 ± 0.016 |  | 0 | 0 | 0 |
| 27 | Isorhamnetin 3-O-rutinoside | 0 | 0 | 0 | 0 | 0 |  | 0.0223 | 0 | 0.011 ± 0.016 |
| 28 | Naringenin 7-O-neohesperidoside (Naringin) | 0.0488 | 0.1336 | 0 | 0 | 0.046 ± 0.063 |  | 0.0625 | 0 | 0.031 ± 0.044 |
| 29 | Quercetin 3-O-rutinoside (Rutin) | 0.0475 | 0 | 0 | 0 | 0.012 ± 0.024 |  | 7.9872 | 0 | 3.994 ± 5.648 |
| 30 | Quercetin 3-O-galactoside (Hyperoside) | 0.0425 | 0.0643 | 0.0317 | 0 | 0.035 ± 0.027 |  | 0.0528 | 0 | 0.026 ± 0.037 |
| 31 | Quercetin 3-O-rhamnoside (Quercitrin) | 0.0938 | 0.1545 | 0.1821 | 0.0531 | 0.121 ± 0.058 |  | 1.1268 | 0.0633 | 0.595 ± 0.752 |
|  | **Sum of glycosides** | **0.276** | **0.457** | **0.276** | **0.053** | **0.266 ± 0.165** |  | **9.369** | **0.087** | **4.728 ± 6.564** |
|  | **Sum of polyphenols** | **35.109** | **38.874** | **35.931** | **16.602** | **31.63 ± 10.15** |  | **95.844** | **39.591** | **67.72 ± 39.78** |
|  | TPC (mgGA/kg) | 628.92 | 721.00 | 565.72 | 437.71 | 588.34 ± 118.95 |  | 1496.83 | 711.88 | 1104.35 ± 555.04 |
|  | RSA (%) | 7.72 | 8.78 | 7.39 | 5.85 | 7.44 ± 1.21 |  | 10.25 | 6.43 | 8.34 ± 2.71 |

Mean ± SD - Mean value ± standard deviations (p ≤ 0.05).
