# Supplementary material for: Polyphenol profile of buckwheat honey, nectar and pollen
Source: R Soc Open Sci. 2020 Dec 9;7(12):201576. doi: 10.1098/rsos.201576 (PMC7813236; doi:10.1098/rsos.201576)
Supplement: Figure S1 [file rsos201576supp2.pdf]

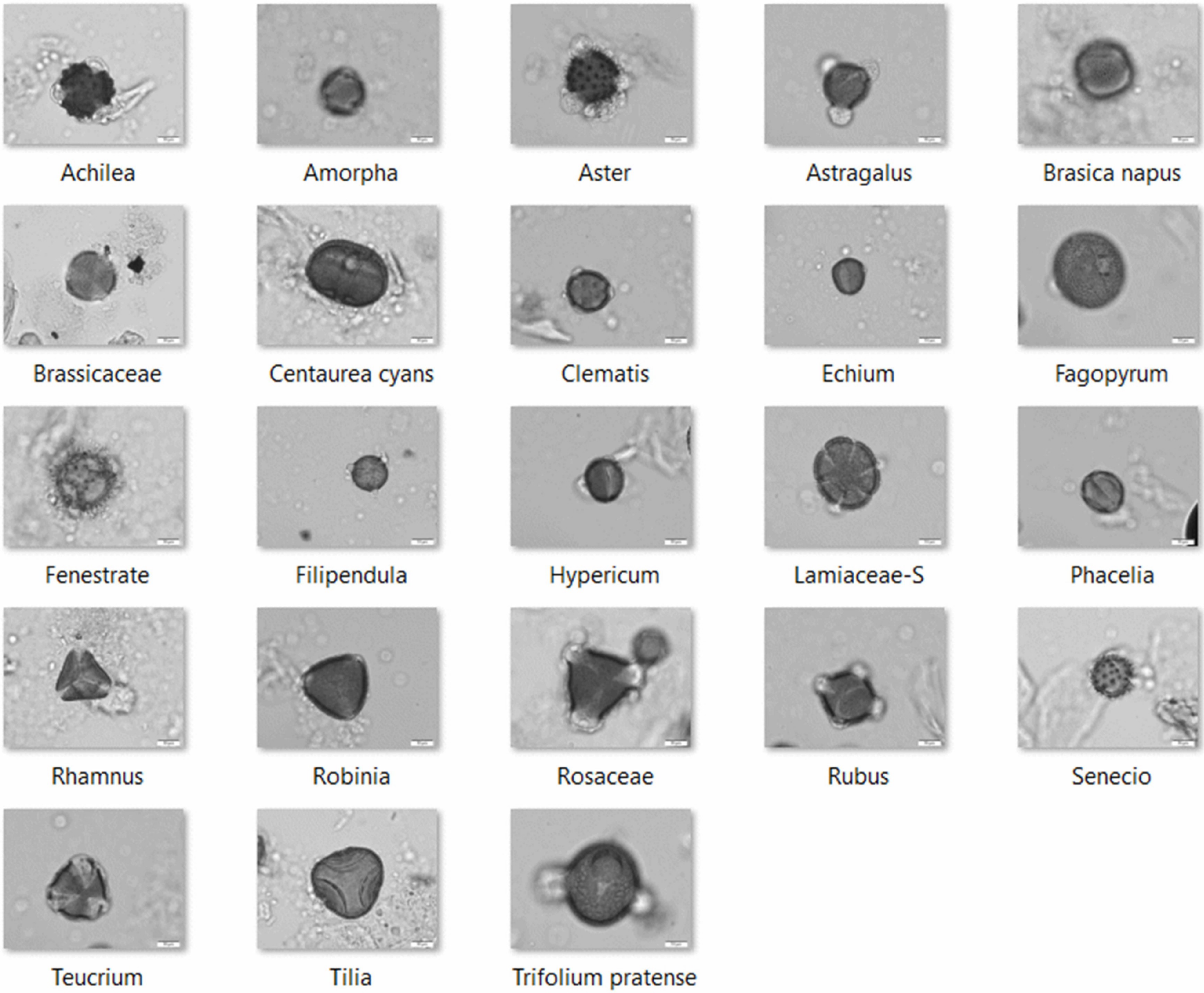
Achilea
Amorpha
Aster
Astragalus
Brasica napus
Brassicaceae
Centaurea cyans
Clematis
Echium
Fagopyrum
Fenestrate
Filipendula
Hypericum
Lamiaceae-S
Phacelia
Rhamnus
Robinia
Rosaceae
Rubus
Senecio
Teucrium
Tilia
Trifolium pratense
